# Comparing the Accuracy of ChatGPT-4o, DeepSeek-V3, and Gemini 2.5 Flash in Answering Frequently Asked Questions About Systemic Lupus Erythematosus: Quantitative Study

**DOI:** 10.2196/89156

**Published:** 2026-06-08

**Authors:** Alvina Widhani, Suzy Maria, Aisha Putri Chairani, Nabila Yulianingrum Adella Visco, Muhammad Faiz Amirullah Nurhadi, Lutfi Airlangga Harjoprawito, Dwitya Elvira, Irwin Tedja, Yuniza Yuniza, Deasy Fetarayani, Sukamto Koesnoe, Bramantya Wicaksana, Anshari Saifuddin Hasibuan, Evy Yunihastuti

**Affiliations:** 1 Division of Allergy and Clinical Immunology, Department of Internal Medicine, Faculty of Medicine, Universitas Indonesia, Diponegoro No. 71 Jakarta, Jakarta, 10430, Indonesia, 62 622-1390-4546; 2 Department of Internal Medicine, Cipto Mangunkusumo General Hospital, Jakarta, Indonesia; 3 Division of Allergy and Clinical Immunology, Department of Internal Medicine, Faculty of Medicine, Andalas University, Padang, West Sumatra, Indonesia; 4 Division of Rheumatology, Allergy-Immunology, Infectious Diseases, Department of Medicine, Persahabatan Hospital, Jakarta, Indonesia; 5 Division of Allergy and Clinical Immunology, Department of Internal Medicine, Faculty of Medicine, Sriwijaya University, Palembang, South Sumatra, Indonesia; 6 Division of Allergy and Clinical Immunology, Department of Internal Medicine, Faculty of Medicine, Universitas Airlangga, Surabaya, East Java, Indonesia; 7 Division of Allergy and Clinical Immunology, Department of Internal Medicine, Dr Soetomo General Academic Hospital, Surabaya, East Java, Indonesia

**Keywords:** chatbot, ChatGPT, Gemini, DeepSeek, artificial intelligence, AI, systemic lupus erythematosus, SLE

## Abstract

**Background:**

Systemic lupus erythematosus (SLE) is a complex, fluctuating disease, creating a continuous need for reliable patient information. A prior study concluded that patients with SLE often turn to the internet, including artificial intelligence (AI) chatbots, for information regarding SLE. The rise of AI chatbots as a primary information source presents a critical challenge regarding the accuracy of the information they provide.

**Objective:**

This study aimed to evaluate the performance of the latest generation of AI chatbots (ChatGPT-4o, DeepSeek-V3, and Gemini 2.5 Flash) in answering frequently asked questions about SLE.

**Methods:**

Twenty-two frequently asked questions about SLE in Bahasa Indonesia (the Indonesian language) were posed to each chatbot. Responses were independently and blindly evaluated for accuracy by 5 clinical immunologists using a 4-point Likert scale. Readability was assessed using the Flesch reading ease score formula. Statistical comparisons for accuracy and readability were performed using repeated-measures ANOVA or the Friedman test, followed by the Bonferroni test for pairwise comparisons. The Spearman ρ was used to evaluate correlations among accuracy, readability, and word count.

**Results:**

Gemini 2.5 Flash demonstrated the highest accuracy, with a mean score of 1.25 (SD 0.53), significantly outperforming ChatGPT-4o (mean 1.71, SD 0.61; *P*<.001). Gemini 2.5 Flash significantly outperformed ChatGPT-4o in 2 evaluated domains. The interreliability analysis revealed a statistically significant level of agreement among the 5 evaluators across all responses (Kendall *W*=0.389; *P*<.001). Readability for all 3 chatbots was low (median Flesch reading ease score 42.22‐46.66). Gemini 2.5 Flash produced the longest responses (8509 total words), followed by DeepSeek-V3 (5410 words) and ChatGPT-4o (3632 words). A significant negative correlation was found between word count and lower accuracy (ρ=−0.401; *P*=.001).

**Conclusions:**

Our study found that ChatGPT-4o, DeepSeek-V3, and Gemini 2.5 Flash provided overall satisfactory responses to SLE-related questions. The highest accuracy was demonstrated by Gemini 2.5 Flash; however, the absolute differences in scores among the 3 AI chatbots were relatively small. All 3 AI chatbots demonstrated low readability, which may limit accessibility for patient use. This finding highlights a critical “blind spot” in which clinical accuracy, as rated by experts, does not equate to patient accessibility. Thus, further research is required to develop more comprehensive evaluation frameworks incorporating safety, factuality, and calibration of AI chatbots across different medical fields and topics.

## Introduction

The rapid advancement of artificial intelligence (AI), particularly in the form of chatbots powered by large language models (LLMs), has changed how the public accesses information. One of the most significantly impacted sectors is health care. These AI chatbots offer many advantages, including 24×7 accessibility, anonymity, and free features, making them an increasingly popular choice for seeking health information [1]. However, this convenience also comes with a critical challenge: ensuring that the information provided is accurate, clinically validated, and safe, rather than potentially misleading or harmful [2,3].

Systemic lupus erythematosus (SLE) is a complex, chronic, multisystem autoimmune disease characterized by a broad spectrum of symptoms that can affect multiple organ systems. The global incidence of SLE is estimated at 5.14 per 100,000 person-years [3]. In Indonesia, the prevalence of SLE is estimated at 0.5% of the population, with more than 1.3 million people affected. Given the growing number of cases and the fluctuating nature of the disease, there is a continuous need for reliable and accessible information regarding symptom management, treatment of side effects, and lifestyle adjustments. A study revealed that 96.9% of patients with SLE search for information about their condition on the internet [4]. Data from 2024 indicated that 83.6% of Indonesians were familiar with AI and used it in their daily activities, predominantly for information-seeking purposes [5,6]. Given the variability in the quality of online information, it is crucial to assess the reliability of AI chatbots in providing accurate responses about SLE.

Prior research has evaluated single chatbots against search engines for SLE-related topics or compared older models across broader autoimmune disease categories. To date, no comparative study has evaluated the performance of the latest generation of AI chatbots in answering frequently asked questions (FAQs) about SLE. This study addresses this critical gap by evaluating and comparing 3 prominent AI chatbots from 2023 to 2025: ChatGPT-4o, DeepSeek-V3, and Gemini 2.5 Flash [6]. Thus, this study aimed to compare the accuracy of ChatGPT-4o, DeepSeek-V3, and Gemini 2.5 Flash in answering FAQs about SLE.

## Methods

### Data Collection

**Figure 1.**
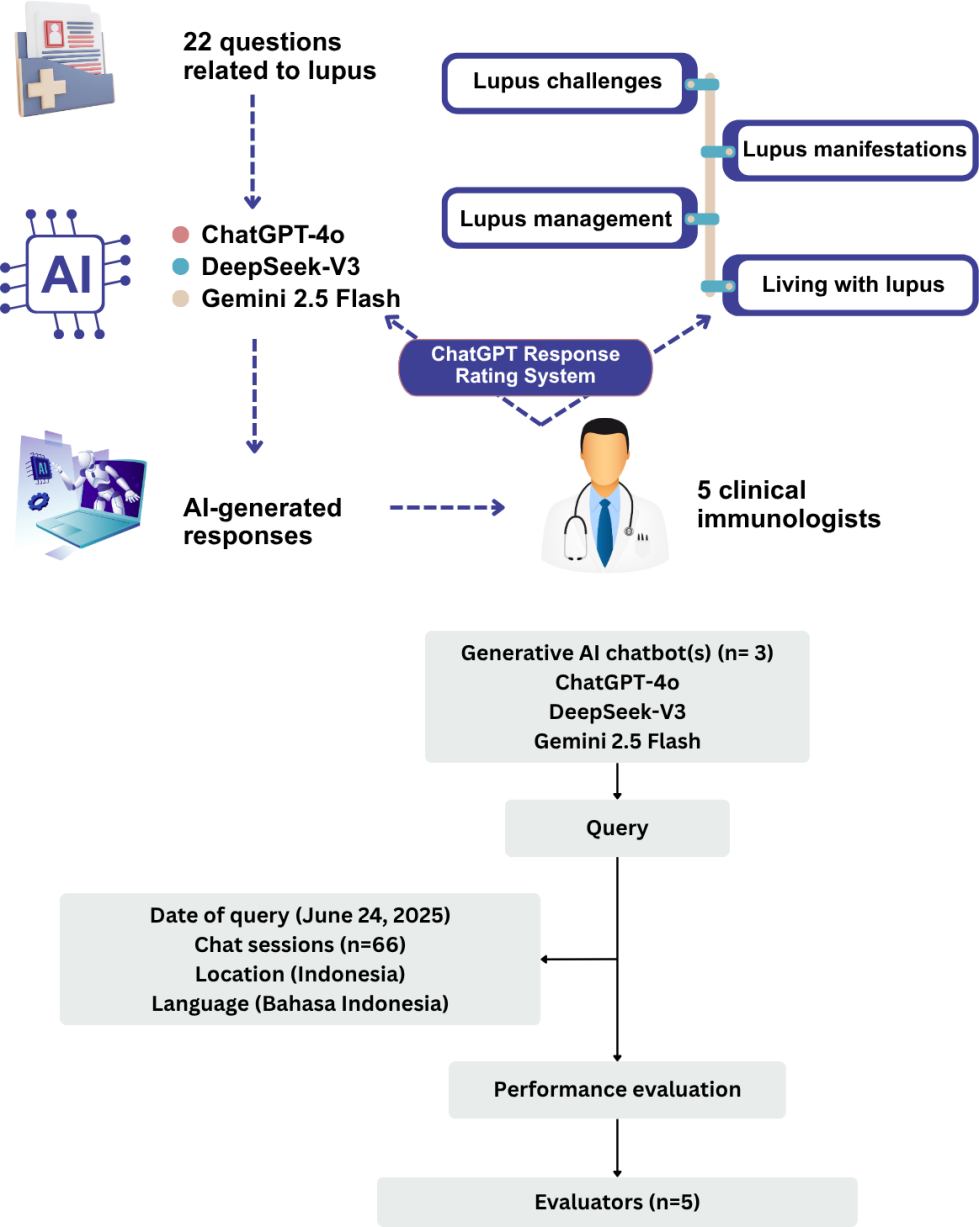
Schematic overview of the overall study design. Five patients with lupus, acting as contributors, ranked 40 questions to identify the 22 most frequently asked questions relevant to patients. These questions were then entered into artificial intelligence (AI) chatbots. Five clinical immunologists evaluated the chatbot responses across 4 domains using the ChatGPT Response Rating System to assess their accuracy.

This study was conducted from May 1, 2025, to September 1, 2025. A schematic overview of the study design is provided in Figure 1. To compile a comprehensive set of FAQs about lupus, 7 authors initially reviewed the FAQ section of the Lupus Canada website and collaboratively developed 40 questions related to SLE [7]. The questions were then divided into the 4 domains listed by lupus100.org: “lupus challenges,” “lupus manifestations,” “lupus management,” and “living with lupus” [8]. This ensured broad coverage of lupus-related inquiries. The questions were then translated into Bahasa Indonesia to align with the study’s target population.

Subsequently, 5 contributors were selected from expert patients with lupus through purposive sampling. They ranked the 40 questions (10 per domain) and were also given the option to add additional questions. All participating patients had been diagnosed with lupus for >5 years and were actively involved in the Indonesian Lupus Foundation (Yayasan Lupus Indonesia). They had roles in patient education and peer counseling through live chats, webinars, and seminars. Because they regularly addressed numerous questions from patients about the disease, these expert patients understood the knowledge gaps faced by the Indonesian population with lupus.

From this input, 4 authors finalized the 20 questions most relevant to patients and individuals without a medical background. The 5 contributors suggested 2 questions that were more common and often raised by patients with lupus. These questions were classified into the “lupus management” domain. The total of 22 questions is provided in Textbox 1. These questions were then individually posed in Bahasa Indonesia into 3 distinct AI chatbot platforms: ChatGPT-4o (OpenAI), DeepSeek-V3 (DeepSeek), and Gemini 2.5 Flash (Alphabet Inc). The AI chatbots’ free online services for consumers were used during a single session on June 24, 2025.

Textbox 1. **Common patient queries regarding systemic lupus erythematosus (commonly referred to as lupus), organized by domain.**

**Lupus challenges**

1. What is lupus?
2. Is lupus a contagious disease?
3. Is lupus dangerous?
4. Who can get lupus?
5. Are there different types of lupus?
**Lupus manifestations**
6. What are the symptoms of lupus?
7. Is a butterfly rash on the face always a sign of lupus?
8. How is lupus diagnosed?
9. I have pain all over my body; could it be caused by lupus?
10. What are the early signs of lupus?
**Lupus management**
11. What therapies are available for lupus?
12. Are there anti-inflammatory foods that can help patients with lupus?
13. What type of diet is recommended to prevent lupus flare-ups?
14. Are there any beneficial supplements for patients with lupus?
15. How can I reduce joint pain caused by lupus?
16. What foods should patients with lupus avoid?
17. Will taking lupus medication for a long time damage the kidneys?
**Living with lupus**
18. Can people with lupus get pregnant?
19. Can lupus be cured?
20. Are patients with lupus not allowed to be exposed to sunlight at all?
21. Is a gluten-free diet beneficial for lupus?
22. How can I manage lupus symptoms and improve my quality of life?

To simulate typical patient behavior, no prompt engineering was used (eg, “Act as a doctor”). The questions outlined in Textbox 1 were entered directly as simple, stand-alone queries. Furthermore, because the study used consumer-facing web platforms rather than application programming interfaces, parameter adjustments such as “temperature” (which controls the randomness of generated text) could not be manually configured. Thus, the responses represent the default, unadjusted outputs generated by each platform’s proprietary algorithms at that specific time. Each question was entered in a new chat box to prevent potential influence from previous queries. The process of the question-and-answer session is presented in both Bahasa Indonesia and English in Multimedia Appendix 1.

### Outcomes

The responses generated by ChatGPT-4o, DeepSeek-V3, and Gemini 2.5 Flash were independently and blindly sent to 5 clinical immunologists for evaluation. All evaluators were lupus experts from tertiary health care centers across Indonesia who had at least 5 years of experience and were actively managing >100 patients with lupus per month. All 5 clinical immunologists received a standardized briefing document consisting of the study objectives, the rating instrument (ChatGPT Response Rating System developed by Mika et al [9]), and the definitions of each scale point. The evaluators critically graded the accuracy of the 22 responses provided by each AI chatbot using established international reference guidelines, including those from the American College of Rheumatology and the European Alliance of Associations for Rheumatology [10-13]. Responses were evaluated using a 4-point Likert scale to assess the clarity and accuracy of each chatbot response. A score of 1 was assigned to excellent responses that did not require clarification, a score of 2 indicated a satisfactory response that required minimal clarification, a score of 3 represented a satisfactory response requiring moderate clarification, and a score of 4 was assigned to a response considered unsatisfactory and requiring substantial clarification. A lower total score indicates higher accuracy and clarity [9].

The readability of each chatbot’s response was assessed using the Flesch reading ease score (FRES) formula [14]. This formula determines textual complexity based on sentence length and syllable count. The FRES formula uses adjusted coefficients: FRES=206.835−(1.0×average sentence length)−(60.0×average syllables per word). The adjustment of the β coefficient to 60.0 accounts for the fact that the syllable count of Indonesian texts must be multiplied by 0.6, given that the ratio of the number of Indonesian and English vocabularies is 1:6 [15,16]. The scoring system has a maximum value of 100, with higher scores indicating greater readability. A score between 60 and 70 is widely considered an acceptable reading level (standard) [17]. The FRES for each answer generated by the chatbots was calculated, averaged, and subsequently compared with the other AI chatbots to evaluate differences in content readability quality.

### Statistical Analysis

The accuracy and readability of the chatbot responses were assessed by calculating the total score from the ChatGPT Response Rating System and the FRES. The mean score and SD for each chatbot were calculated using SPSS (version 27.0; IBM Corp). To determine the significance of mean differences in accuracy scores (Likert scale) and FRES, repeated-measures ANOVA was conducted if the data demonstrated a normal distribution. If the data did not display a normal distribution, the Friedman test was used, followed by the Bonferroni correction for subsequent pairwise comparisons. Results were then illustrated using GraphPad Prism (version 10.1.0; GraphPad).

To investigate the relationship between the characteristics of the AI responses, a Spearman ρ correlation analysis was performed. This analysis aimed to determine the strength and direction of the relationships among the following 3 metrics: the expert-rated mean score (accuracy), the number of words (length), and the FRES (readability). Interrater reliability among the 5 clinical immunologists was evaluated using the Kendall *W* coefficient of concordance to appropriately account for the ordinal nature of the 4-point Likert scale. Spearman ρ was chosen because of the ordinal nature of the Likert scale data and the nonnormal distribution observed in the other metrics. Results were considered statistically significant at *P*<.05.

### Ethical Considerations

This study was approved by the ethics committee of the Faculty of Medicine Universitas Indonesia–Cipto Mangunkusumo Hospital (KET-1296/UN2-F1/ETIK/PPM.00.02/2025). The 3 AI chatbots used are publicly accessible platforms; therefore, no permission was required to use the information generated. All 5 patients with lupus who participated in the question-ranking process provided written informed consent prior to participation. The consent form explained (1) the study purpose and procedures; (2) that participation was voluntary and could be withdrawn at any time without consequence; (3) that no clinical data, identifying information, or health records would be collected; (4) that their contributions would be acknowledged only with explicit permission, as listed in the Acknowledgments section; and (5) that no monetary compensation would be provided.

## Results

### Accuracy of Information

**Figure 2.**
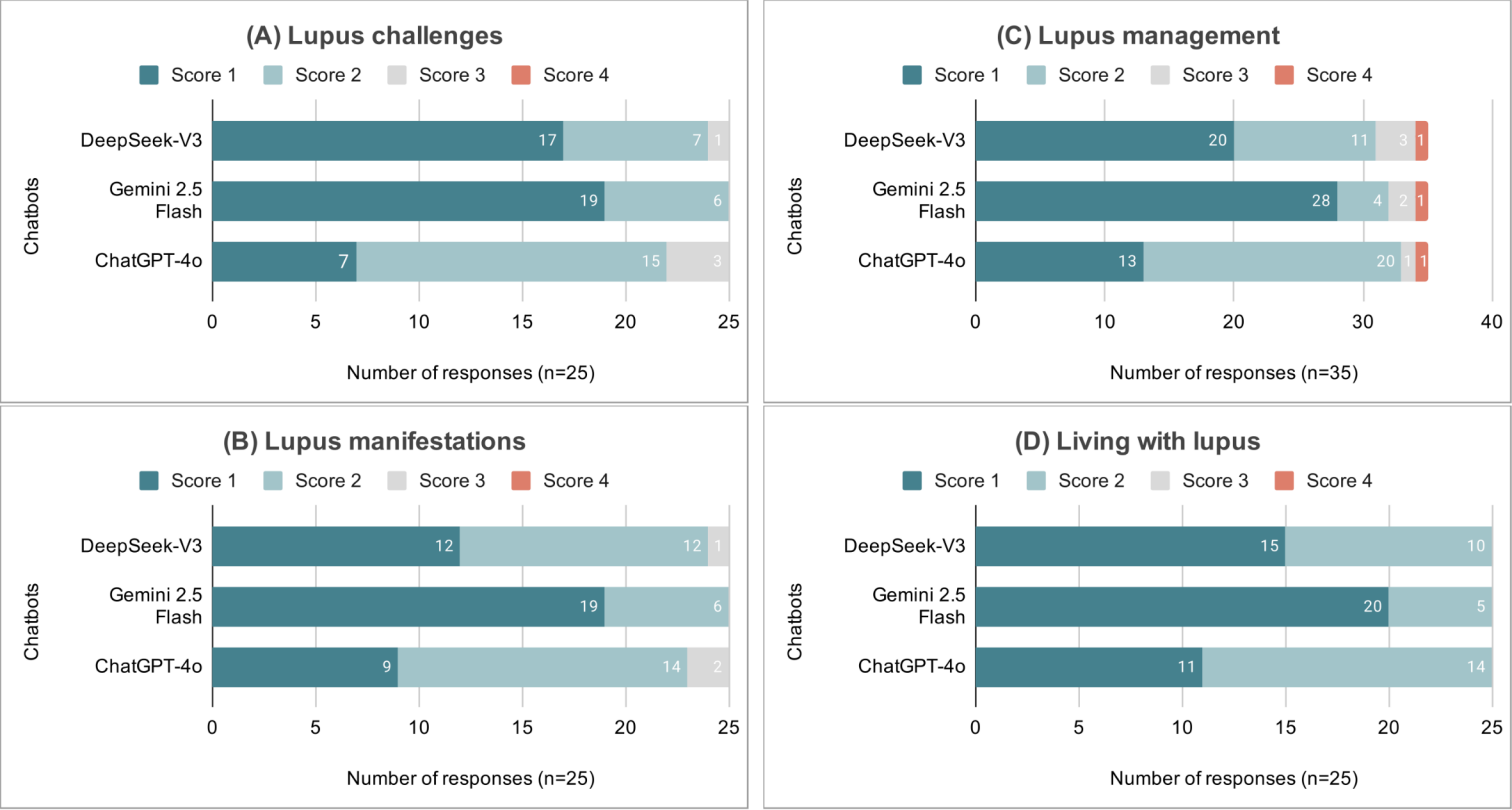
Number of each score given by experts compared to the total number of responses in each domain. (A) Lupus challenges, (B) lupus management, (C) lupus manifestations, and (D) living with lupus. The 4-point Likert scale was defined as follows: 1=“excellent response not requiring clarification,” 2=“satisfactory response requiring minimal clarification,” 3=“satisfactory response requiring moderate clarification,” and 4=“unsatisfactory response requiring substantial clarification.”

Gemini 2.5 Flash had the best average rating per question, with a mean score of 1.25 (SD 0.53), followed by DeepSeek-V3 with a mean score of 1.48 (SD 0.63) and ChatGPT-4o with a mean score of 1.71 (SD 0.61; *P*<.001), as shown in Table 1. This indicates generally accurate and comprehensive responses from all 3 chatbots. The total number of responses was 110 from 22 questions evaluated by 5 evaluators. Of the 110 responses, 8 (7.3%) responses from Gemini 2.5 Flash had the same score as the highest-rated answer, having been given a perfect mean score of 1 (SD 0) from all 5 reviewers (Q6, Q10, Q11, Q12, Q14, Q16, Q20, and Q22), with most answers given a score of 1 (n=86, 78.2%). The score breakdown by domain is shown in Figure 2. Each model received the lowest score of 4 from the same expert for the question “How can I reduce joint pain caused by lupus?” in the “Lupus Management” domain. DeepSeek-V3’s answer to the question “What type of diet is recommended to prevent lupus flare-ups?” obtained the worst mean score among all graded responses (mean 2.4, SD 0). Despite this finding, DeepSeek-V3 received perfect mean scores of 1 (SD 0) for 2 questions, and a substantial number of responses were assigned a score of 1 (n=64, 58.2%). ChatGPT-4o received no perfect mean scores, with most evaluations given a score of 2 (n=63, 57.3%) and a substantial proportion given a score of 1 (n=40, 36.4%).The score breakdown by domain is shown in Figure 2. Each model received the lowest score of 4 from the same expert for the question “How can I reduce joint pain caused by lupus?” in the “Lupus Management” domain. DeepSeek-V3’s answer to the question “What type of diet is recommended to prevent lupus flare-ups?” obtained the worst mean score among all graded responses (mean 2.4, SD 0). Despite this finding, DeepSeek-V3 received perfect mean scores of 1 (SD 0) for 2 questions, and a significant number of responses were assigned a score of 1 (n=64, 58.2%). ChatGPT-4o received no perfect mean scores, with most evaluations given a score of 2 (n=63, 57.3%) and a substantial proportion given a score of 1 (n=40, 36.4%). Interrater reliability among the 5 immunologists was assessed using the Kendall *W* coefficient of concordance. The overall Kendall *W* was 0.389 (*P*<.001), indicating a statistically significant, fair-to-moderate level of agreement. Kendall *W* was used as it accounts for the ordinal nature of the Likert-based scoring system [18]. Detailed scores for each question are provided in Multimedia Appendix 2.

**Table 1.** Mean scores, word counts, and Flesch reading ease score (FRES) values across the 3 artificial intelligence chatbots^a^.

Variables	DeepSeek-V3	Gemini 2.5 Flash	ChatGPT-4o	*P* value
Overall score, mean (SD)	1.48 (0.63)	1.25 (0.53)	1.71 (0.61)	<.001^b^
Sum of scores	122	106	156	<.001
Score distribution by domain, mean (SD)				
Domain 1	1.36 (0.57)	1.24 (0.44)	1.84 (0.62)	<.001^b^
Domain 2	1.56 (0.58)	1.24 (0.44)	1.72 (0.61)	.007^b^
Domain 3	1.57 (0.78)	1.31 (0.72)	1.71 (0.67)	.001^b^
Domain 4	1.4 (0.5)	1.2 (0.41)	1.56 (0.51)	.03^b^
Word count, mean (SD)	245.91 (68.85)	386.77 (149.08)	165.09 (71.55)	<.001^c^
Sum of words	5410	8509	3632	<.001
FRES, median (IQR)	46.66 (39.38-47.71)	42.22 (35.53-47.31)	46.19 (39.10-53.53)	.01^b^

a Interrater reliability among the 5 clinical immunologists was fair to moderate (Kendall *W*=0.389; *P*<.001).

b Friedman test.

c Repeated-measures ANOVA.

**Figure 3.**
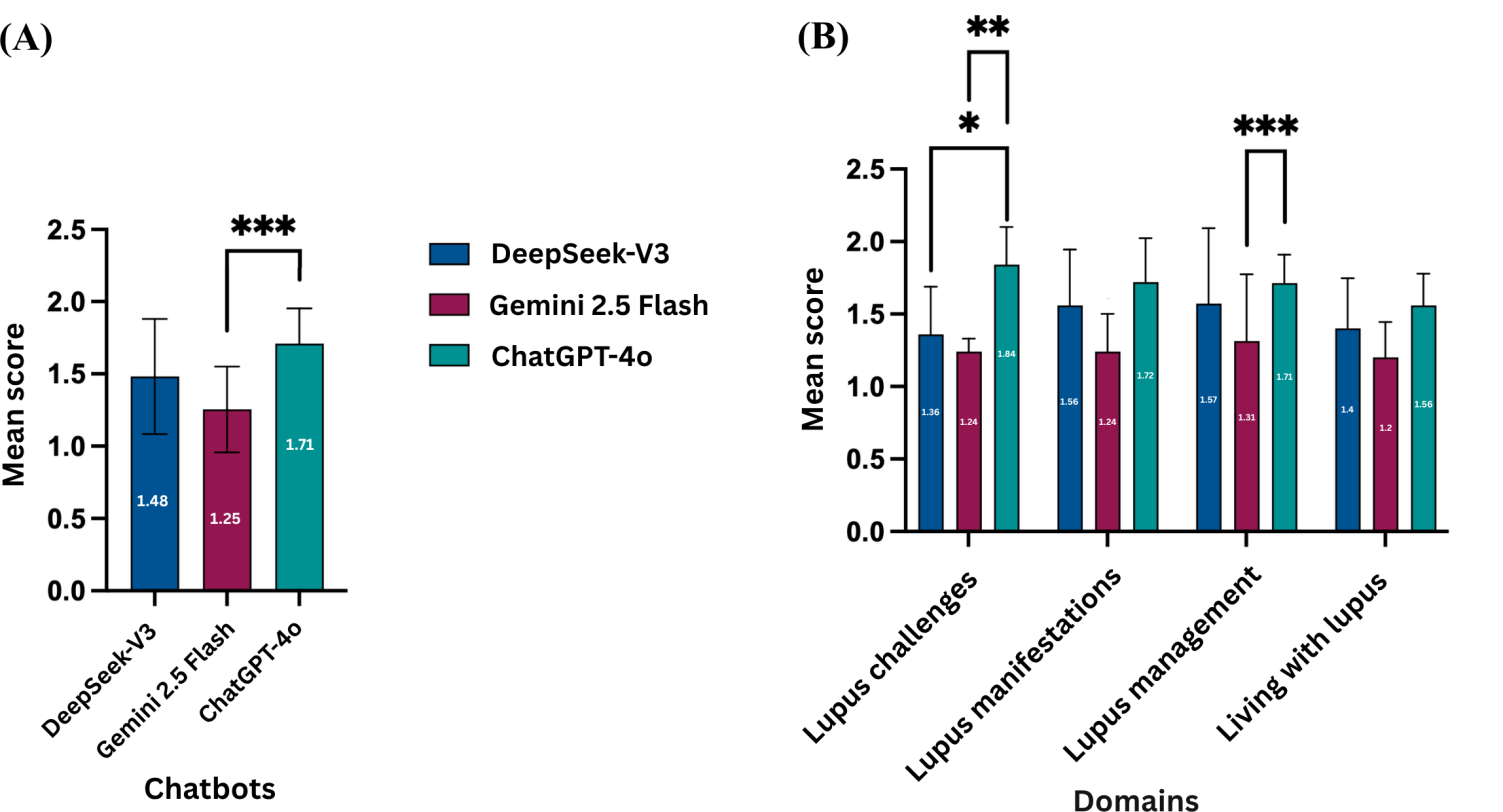
Mean expert scores for chatbot responses. (A) Overall mean scores for DeepSeek-V3, Gemini 2.5 Flash, and ChatGPT-4o and (B) multiple comparisons of the mean scores for the 4 domains: lupus challenges, lupus manifestations, lupus management, and living with lupus. Scores range from 1 to 4 based on expert evaluations. The bar graphs represent mean scores, with error bars indicating the SDs. *P<.05, **P≤.01, and ***P≤.001.

The Bonferroni correction showed that Gemini 2.5 Flash had a significantly higher mean score than ChatGPT-4o (*P*<.001), as illustrated in Figure 3. Although there were significant differences in mean scores across all domains, as shown in Table 1, Gemini 2.5 Flash only outperformed ChatGPT-4o in answering questions from 2 domains: “Lupus challenges” and “Lupus management.” All 3 AI chatbots advised users to consult a physician for further evaluation and management if symptoms were present.

### Readability

Readability of the responses was assessed using the FRES. Across all 3 AI chatbots, the mean FRES values were below the recommended standard of 60. The median FRES values for DeepSeek-V3, Gemini 2.5 Flash, and ChatGPT-4o were 46.66 (IQR 39.38-47.71), 42.22 (IQR 35.53-47.31), and 46.19 (IQR 39.10-53.53), respectively. This indicates that the responses generated by all 3 chatbots were considered to require a college graduate reading level and were therefore difficult to read. The Friedman test confirmed that the FRES differed significantly across the 3 models (*P*=.01). Subsequent pairwise comparisons indicated that the only significant difference in readability was observed between Gemini 2.5 Flash and DeepSeek-V3 (*P*=.01). A disparity was also observed in word count, with Gemini 2.5 Flash having the highest word count of 8509 words, followed by DeepSeek-V3 with 5410 words and ChatGPT-4o with 3632 words. The Bonferroni correction also showed that the mean word counts of all models differed significantly. The word count and FRES for each model are summarized in Table 2.

To investigate the relationship between the characteristics of the responses and their accuracy, a nonparametric Spearman ρ correlation analysis was conducted across the 3 primary metrics. The analysis revealed a significant negative correlation between the expert-rated mean score and the number of words (ρ=−0.401; *P*=.001). Meanwhile, the FRES showed no statistically significant correlation with either the mean score (ρ=0.218; *P*=.08) or the number of words (ρ=−0.202; *P*=.10).

**Table 2.** Word count and Flesch reading ease score (FRES) for responses generated by DeepSeek-V3, Gemini 2.5 Flash, and ChatGPT-4o.

Domains and questions^a^	Word count			FRES		
	DeepSeek-V3	Gemini 2.5 Flash	ChatGPT-4o	DeepSeek-V3	Gemini 2.5 Flash	ChatGPT-4o
Lupus challenges						
Q1	183	366	104	45.715	47.305	53.525
Q2	112	58	39	45.385	42.185	46.435
Q3	223	324	80	46.485	43.405	39.185
Q4	206	229	126	44.225	44.225	46.765
Q5	229	260	148	46.745	42.215	46.665
Lupus manifestations						
Q6	283	447	165	45.705	47.205	47.645
Q7	136	267	93	46.785	42.215	45.895
Q8	303	559	243	46.645	42.725	45.635
Q9	236	442	93	47.655	46.675	39.105
Q10	291	301	160	47.705	46.565	46.695
Lupus management						
Q11	282	504	173	39.605	42.215	45.625
Q12	296	540	138	39.375	41.355	45.935
Q13	291	636	344	46.695	40.325	46.685
Q14	327	560	179	46.695	35.525	42.125
Q15	215	429	285	46.685	42.125	46.685
Q16	217	470	142	46.695	40.325	42.295
Q17	295	341	200	44.355	44.875	43.695
Living with lupus						
Q18	219	368	172	45.355	42.215	41.105
Q19	157	106	111	46.665	46.685	46.665
Q20	195	342	213	46.695	43.405	46.565
Q21	313	389	170	43.405	41.105	42.125
Q22	401	571	254	46.695	42.125	46.675

a Please refer to Textbox 1 for the full questions.

## Discussion

### Principal Findings

All 3 chatbots provided satisfactory responses to lupus-related questions. Gemini 2.5 Flash demonstrated the highest accuracy compared to DeepSeek-V3 and ChatGPT-4o, with its mean score significantly outperforming ChatGPT-40 and showing superior performance across 2 evaluated domains: “lupus challenges” and “lupus management.” This suggests that the model possesses a highly organized and comprehensive knowledge base and is equally capable of answering questions about clinical symptoms, lifestyle, and patient experience. Gemini 2.5 Flash’s significantly higher word count allowed more detailed answers, which was supported by the significant negative correlation between the expert-rated mean scores and word counts, indicating that longer responses were generally associated with better evaluations (lower mean scores) from the clinical immunologists.

Furthermore, readability remains a fundamental concern in the development of AI-generated medical content. All 3 AI models produced text with similarly low FRES values of <50, indicating a “difficult to read” level generally reserved for college-graduate material. This can be attributed to the medical terminology within the generated responses, despite the questions being structured from a patient’s perspective. There was no significant correlation between readability scores and other factors, indicating that readability is independent of accuracy and response length. This suggests that experts focused mainly on clinical accuracy rather than language accessibility when rating AI chatbot responses.

### Comparison With Prior Work

This consistently low readability finding is in accordance with other studies examining various topics, including appendicitis by Ghanem et al [19], vasectomy by Carlson et al [20], and radiotherapy by Grilo et al [21], which all reported that AI-generated medical information frequently falls below the recommended readability standard for general patient comprehension. The only statistically significant difference in readability was observed when DeepSeek-V3 scored significantly better than Gemini 2.5 Flash (*P*=.002) [18-20]. Assessing chatbot responses about basal cell carcinoma, Sidhu and Selvamogan [22] also found that ChatGPT-4o achieved the highest mean FRES compared with Gemini 2.5, Grok 3, and DeepSeek-R1, even though it was still fairly difficult to read. This finding contradicts trends reported by Ghanem et al [19] and Carlson et al [20], who found the preceding Google Bard model to be the most intelligible.

It is important to acknowledge that, as highlighted by Jindal and MacDermid [23], the FRES formula should not be equated with actual comprehension of user understanding, considering that FRES primarily assesses sentence length and word complexity. Crucial factors such as document design (layout, pictures and charts, color, font, spacing, legibility, and grammar), user characteristics (education level, health literacy, prior knowledge, and anxiety levels), and style of writing (cultural sensitivity and appropriateness) are not considered, despite affecting comprehension [23].

Our findings highlight both the potential of AI chatbots in health care and the significant performance variations among models. Although our study identified Gemini as the superior model for addressing questions about SLE, this outcome is not universal across all medical specialties. For instance, a study on chatbot responses to vasectomy-related questions by Mouhawasse et al [24] found ChatGPT-4o to be more accurate than Gemini (formerly Bard). This discrepancy suggests that the nature of the medical topic is a critical factor. The nuances of a chronic systemic disease such as SLE may present unique challenges for AI models that differ from those posed by more straightforward procedural topics such as vasectomy.

Furthermore, this study’s finding that ChatGPT-4o had a significantly higher mean score than Gemini and DeepSeek is particularly relevant given its widespread use. This suggests that although ChatGPT-4o may provide a baseline level of helpfulness, its accuracy and comprehensiveness for complex medical topics such as SLE may be limited. This finding aligns with other research assessing the accuracy and reliability of ChatGPT-4o in the context of cancer care, which also found the need to carefully evaluate its output [25]. The consistent indication of need for careful evaluation across different medical domains, including SLE, vasectomy, and cancer, underscores the importance of validating AI-generated medical information before it is used by patients or clinicians [24,26].

The differences between accuracy and readability observed in this study reflect a broader challenge in deploying LLMs for public health. Prior studies by Aydin et al [27] and Vishwanath et al [28] underscore the significant potential of LLMs to improve patients’ experiences in health care communication. Additionally, Miftaroski et al [29] and Will et al [30] demonstrate LLMs’ ability to simplify and enhance the readability of patient education materials, making them more accessible and potentially narrowing the gap between patients and health care providers.

However, this study reveals an important relationship between clinical accuracy and readability. Despite the high accuracy confirmed through blinded evaluation by 5 clinical immunologists across 3 AI models, this did not necessarily align with effective multilingual communication in health care. This suggests that although LLMs demonstrate proficiency in generating medically accurate content, they remain inadequate as effective communicators for patients. This gap underscores the need to address language barriers in LLM deployment, as multilingualism is critical for improving health care outcomes [31].

The approach of using a patient-centric method to formulate the questions for the AI chatbots is a notable strength of this study. By involving patients with lupus in the question-gathering process, the study ensured that the topics covered were truly relevant to the patient experience, making the results more applicable to real-world scenarios. This patient-centered approach should be a standard practice in future research on AI in health care.

The ethical implications of AI chatbot use in health care are a central point for discussion. A physician’s role extends beyond interpreting clinical status to encompass the patient’s unique emotions, feelings, and social values—a holistic assessment that AI cannot perform [17]. Therefore, all AI-generated responses should include a disclaimer advising users to consult a health care professional, advice that was given by most of the chatbots examined in this study. The findings of this study reinforce this necessity, as even the best-performing model in this study, Gemini, is fallible. In a study by Barlas and Tunç [25], chatbots provided sufficient responses to general descriptive questions but also produced insufficient and misleading responses [32]. In the context of chronic diseases such as SLE, patient education and the delivery of reliable information are important to ensure that patients receive accurate and trustworthy guidance.

A significant area for future research is expanding on the findings of this study by exploring the performance of AI models across a broader range of languages. As this study was conducted only in Bahasa Indonesia, it is important to investigate whether the findings are generalizable to other languages. A study by Ando et al [33] examined the quality of ChatGPT responses in English and Japanese and found that English responses scored higher than Japanese responses. Furthermore, future research should examine the effects of various prompting strategies, as highlighted by Wang et al [34], who reported that differences in prompts can affect the accuracy of AI responses. By continuing to refine and validate these studies, the health care community can responsibly harness the potential of AI to improve patient education and support.

### Limitations

This study has several limitations. First, it used a single-pass evaluation using consumer web interfaces. As LLMs are nondeterministic, they often generate different responses to the same prompt. Without application programming interface access to control temperature settings or run multiple trials, reproducing the exact text is difficult. Nevertheless, the general public would have access to this form of AI chatbot. Thus, variability in AI chatbot responses is part of the real-time user experience. Second, the rating system adopted by Mika et al [9] may not fully assess a high-quality response. Future research should explore more comprehensive approaches, as suggested by Ma et al [35], using a 6-aspect rating system that includes safety, simplicity, relevance, and completeness to provide a better understanding of how AI models perform across different dimensions of quality, a crucial aspect for patient care. Finally, agreement among raters was fair to moderate (Kendall *W*=0.389), reflecting variability in expert assessments. Greater disagreement was observed in responses related to dietary management in SLE, an area in which applicable clinical guidance remains limited. In such contexts, evaluators may rely more heavily on individual clinical experience, which can contribute to differences in scoring. In addition, further studies regarding safety, factuality, and calibration are required to increase credibility and provide a standardized methodology when conducting studies that evaluate AI chatbot responses.

### Conclusions

This study compared the performance of 3 latest-generation AI chatbots in answering FAQs about SLE. Our study found that ChatGPT-4o, DeepSeek-V3, and Gemini 2.5 Flash provided overall satisfactory responses to SLE-related questions. The highest accuracy was demonstrated by Gemini 2.5 Flash; however, the absolute differences in scores among the 3 AI chatbots were relatively small. All 3 AI chatbots in this study showed low readability. The consistently “difficult to read” level observed across platforms may limit their accessibility for patient use. This study’s key strength of using expert immunologists also revealed an important “blind spot,” as the scoring system failed to reflect the substantial gap between clinical accuracy and patient understanding.

Future research should incorporate comprehensive evaluation frameworks, including safety, factuality, and calibration, to improve the credibility of AI chatbot assessments. Despite the benefits that AI chatbots offer, their use in health care must always be accompanied by regular clinical follow-up and guidance from physicians to prevent misinformation and ensure safe decision-making.

## Supplementary material

10.2196/89156 Multimedia Appendix 1 Questions and chatbot responses in Bahasa Indonesia and English.

10.2196/89156 Multimedia Appendix 2 Detailed ratings from individual evaluators for each question.

## Abbreviations

AI: artificial intelligence
FAQ: frequently asked question
FRES: Flesch reading ease score
LLM: large language model
SLE: systemic lupus erythematosus
